# Editorial: Methods and applications in integrative physiology

**DOI:** 10.3389/fphys.2022.1096216

**Published:** 2022-11-30

**Authors:** Ovidiu Constantin Baltatu, Chao-Yin Chen, Guido Caluori

**Affiliations:** ^1^ Center of Innovation, Technology and Education (CITE) at Anhembi Morumbi University—Anima Institute, Sao Jose dos Campos, Brazil; ^2^ College of Medicine and Health Sciences, Khalifa University, Abu Dhabi, United Arab Emirates; ^3^ Department of Pharmacology, University of California, Davis, Davis, CA, United States; ^4^ IHU LIRYC, Electrophysiology and Heart Modeling Institute, Fondation Bordeaux Université, Pessac, France; ^5^ University of Bordeaux, INSERM U1045, Cardiothoracic Research Center of Bordeaux, Pessac, France

**Keywords:** data collection, data analysis, technology, computational biology, computer simulation

Physiology is the science of function in living organisms that serves as the foundation of knowledge for clinical and experimental medicine. Physiology is an emerging property of tissues and organs. This definition gives us a direct feeling of its complexity and of the multifaceted approaches to investigating it.

Integrative physiology is concerned with the larger elements of physiology that entail the integration of processes and regulatory functions at all biological levels, from the molecular, cellular, tissue, and organ levels to clinical research. Integrative physiology utilizes knowledge from multidisciplinary research that blends life sciences and mathematical sciences. Therefore, its methodology—as in the procedures or strategies used to find, select, process, and evaluate evidence addressing a specific question—is often multi- and cross-disciplinary. Since knowledge in integrative physiology, clinical and experimental medicine is constantly expanding, so too are the methodologies and strategies to approach its questions. New or adapted technologies are the key to enable and expand these needed advancements.

This Research Topic is part of the Methods and Applications in Physiology series. This series showcases the recent advances in experimental techniques and methods used to understand the most fundamental questions in physiological research, ranging from molecular to organ function in living organisms. This Research Topic focuses on cutting-edge emerging technologies and techniques for new sensors, data gathering, processing, analysis, and modeling for various academic, clinical, and healthcare applications ranging from bench to bedside.

We, as Guest Editors, are pleased to present 13 papers and are grateful for the 74 contributions from 46 institutions in 11 countries. This Research Topic of articles includes the use of new methodologies for evaluating physiological (Hillman et al.; Liu et al., 2022; Luck et al.; Švecová et al.) and pathological (Lu et al.; Odehnalová et al.) processes, how computational approaches can provide new insights into how molecular events leading to physiological behavior and guide the future wet bench research (Gerach et al.; Gionti et al.; Gurel et al.; Yoneda et al.), the benefits and disadvantages of methods in integrative physiological research (Horváth et al.; Stracina et al.) and the species difference in autonomic regulation (Bazmi and Escobar). We would like to introduce, for interested readers, the synopsis of each accepted contribution:

## Cardiac imaging

Gross pathology examination is the gold standard for morphological evaluation of focal myocardial disease. The 9.4 T magnetic resonance imaging (MRI) is a new approach for visualizing myocardial pathology. Using an experimental radiofrequency (RF) ablation lesion in swine heart tissue, Odehnalová et al. demonstrated that lesions can be measured on high-resolution MRI images with the same accuracy as on pathological sections and compared these two methods for the evaluation of experimental radiofrequency (RF) ablation lesion in swine heart tissue.

## Laser doppler flowmetry

Laser Doppler flowmetry (LDF) probes, which provide continuous, non-invasive assessment of skin blood flow, are routinely used to quantify cutaneous microcirculatory perfusion. When an LDF probe is removed and replaced, as is the case during pre- and post-intervention or between-day assessments, inhomogeneities in the skin’s microvasculature density contribute to a deterioration in reproducibility. Luck et al. demonstrated that increasing the number of individual LDF probes in a custom-made holder improves LDF measurement reproducibility during test retests. These findings imply that cutaneous vascular conductance (CVC) measurements acquired with numerous laser Doppler probes (number ≥4) have a sufficiently high degree of repeatability after the probes are replaced within the same individual. As a result, we propose that the results from numerous laser Doppler probes in a holder be used in the intraindividual analysis for investigations in which the LDF probes must be removed and replaced in the same participants.

## New method to estimate both the surface and t-tubular capacitance/area in cardiomyocytes

Švecová et al. have devised a new method for determining the fraction of t-tubular membrane by perfusing the tested cell with an isotonic solution with low conductivity. It can repeat measurements on the same cardiomyocyte, allowing for paired statistical testing, which is an advantage over previous approaches. The procedure is straightforward and noninvasive for the measured cell, and it can be applied to investigate short-term alterations in the t-tubular system.

## A simulation model for investigating stretch activation in heart pumping

Yoneda et al. created a Monte Carlo (MC) cross-bridge model with a trapping mechanism to shed light on the role of stretch activation in heart pumping. In this Monte Carlo cross-bridge model, they included a mechanism for trapping the myosin molecule in its post-power stroke state. Yoneda et al. calculated the rate constants of transitions for trapping and escape in a thermodynamically consistent manner. They derive the following findings about the stretch activation process based on our numerical analysis: Because the population of trapped myosin molecules and their average force increase after stretching, the delayed force becomes larger than the original isometric force; ii) the delayed force has a duration of more than a few seconds due to a relatively small rate constant of escape from the trapped state.

## Goldfish autonomic nervous system experimental model

Bazmi and Escobar examined ventricular action potentials, electrocardiograms, and Ca^2+^ transients from perfused Goldfish intact hearts with either a sympathetic or parasympathetic agonist to determine how stimulation of either autonomic nervous system branch impacted cardiac contractility and excitability. Their findings show that stimulating the Goldfish autonomic nervous system with these regularly used agonists resulted in a commensurate alteration in heart dromotropism, chronotropism, inotropism, and lusitropism, like what has been reported in humans. Although the Goldfish heart only has two chambers, the authors conclude that its similar electrical and autonomic features make it a valuable model for studying larger mammalian pathophysiology.

## In-silico model for left ventricular dyssynchrony and impaired torsion

Gerach et al. investigated the specific hypothesis that dyssynchrony alone affects the kinetics of the left ventricle in patients with heart failure with reduced ejection fraction (HFrEF) and left bundle branch block (LBBB) in such a way that rotational behavior is qualitatively altered. If this was the case, they would have been able to replicate the mechanical behavior found in the NOGA XP cardiac mapping system *in vivo*. The fact that this theory was falsified indicates that it is not the electromechanical activation sequence alone that controls rotational behavior, implying that other mechanisms are involved. The implications include that more research is needed to fully understand the drivers of rotating behavior and that these additional (and now unknown) mechanisms are likely equally crucial to address therapeutically.

## Calcium transient high-throughput assay for the detection of acute cardiac hazard in early drug discovery

Lu et al. developed an acute cardiac hazard score system applied to the Ca^2+^ transient assay using different human-induced pluripotent stem cell-derived cardiomyocytes (hiPSC-CM) cell lines to easily identify the concentration-dependent level of cardiac risk by concentration for new chemical entities based on Ca^2+^ transient measurement in hiPSC-CMs using multiple functional parameters including beat rate, calcium transient duration at 90% of repolarization (CTD90), and amplitude. The human-induced pluripotent stem cell-derived cardiomyocytes assay system’s calcium transient high-throughput assay is simple to use and inexpensive.

## Using the action potential clamp technique to investigate cardiac ion channel coordination

Horváth et al. traced the evolution of the voltage clamp technique from its traditional application of the rectangular command to the onion-peeling method. These new methods expand the possibilities for investigating the function of individual ion channels as well as the coordination of membrane currents during AP. The onion-peeling technique is particularly well suited for studying the interaction of currents and thus has a distinct potential in integrative physiology. It was designed for cardiac myocytes but can be applied to skeletal and smooth muscle cells, neurons, and any excitable cell type where ionic currents and AP control cell function.

## New parameters for stratifying arrhythmic risk

Gionti et al. used numerical simulations based on left ventricular models derived from post-myocardial infarction patients to identify some features of proarrhythmic geometric configurations of scars and border zones. A geometric pattern of scar and border zones, characterized by thin subendo- and subepicardial border zones and transmural border zone isthmuses, was identified at cardiac magnetic resonance as a major risk for sustained ventricular tachycardia inducibility at electrophysiology study. This data is simple to apply in daily life and could be combined with known non-invasive risk factors to identify patients with ischemic cardiomyopathy and moderate systolic dysfunction who should undergo an electrophysiology study.

## A review and future outlook on ECG applications in clinical and experimental settings

Stracina et al. reviewed progress and presented perspectives on ECG recording and analysis. Through a multidisciplinary approach, the authors summarize the potential of advanced data analysis. Special emphasis is placed on innovative deep-learning techniques that have been intensively expanded in a wide range of clinical applications and offer promising prospects in experimental branches.

## Cardiac neural network dynamics—opportunities and challenges

The fundamental investigations into the cardiac neural control hierarchy were reviewed by Gurel et al. They discuss the use of computational methods to investigate how information is processed while closed-loop control is in operation and to guide better experimental design. The large cardio-neural datasets produced by these experimental designs necessitate sophisticated signal processing and time series analysis techniques, as well as the usual large-scale computational challenges related to data sharing and reproducibility. These difficulties offer excellent chances for developing and approving cutting-edge methods to improve the understanding of the mechanisms underlying cardiac pathologies needed for clinical application.

## An open-source hypoxia/hyperoxia exposure chamber

Hillman et al. created a simple hypoxia chamber using off-the-shelf components and controlled by open-source software to collect continuous data on oxygen levels and other environmental factors (temperature, humidity, pressure, light, sound, etc.). They created a low-cost and customizable system using FLOS (Free-Libre and Open-Source) software and hardware that can be used for a variety of experimental protocols. This hypoxia/hyperoxia exposure chamber enables reproducible and transparent data acquisition as well as increased consistency with a high degree of customization for each experimenter’s needs.

## Human hypertension blood flow model

Bahloul et al. proposed a fractional model with high flexibility for characterizing the arterial complex tree network. Through a series of validations on human hypertensive patients, the results demonstrate the validity of the new model and the physiological interpretability of the fractional differentiation order. Furthermore, the findings show that the fractional-order modeling approach has a high potential for improving understanding of the structural and functional changes in the large and small arteries caused by hypertension disease.

We gratefully acknowledge the work of the 74 authors of the Frontiers Research Topic (RT) entitled *Methods and Applications in Integrative Physiology*, and eagerly anticipate future discoveries, research, and developments in integrative physiology.
